# Cell Engineering and Molecular Pharming for Biopharmaceuticals

**DOI:** 10.2174/1874104500802010049

**Published:** 2008-05-14

**Authors:** M.A Abdullah, Anisa ur Rahmah, A.J Sinskey, C.K Rha

**Affiliations:** 1 Department of Chemical Engineering, Universiti Teknologi Petronas, Tronoh, Perak, Malaysia; 2 Department of Biology, Massachusetts Institute of Technology, Cambridge, Massachusetts 02139, USA; 3 Biomaterials Science and Engineering Laboratory, Massachusetts Institute of Technology, Cambridge, Massachusetts 02139, USA

## Abstract

Biopharmaceuticals are often produced by recombinant *E. coli* or mammalian cell lines. This is usually achieved by the introduction of a gene or cDNA coding for the protein of interest into a well-characterized strain of producer cells. Naturally, each recombinant production system has its own unique advantages and disadvantages. This paper examines the current practices, developments, and future trends in the production of biopharmaceuticals. Platform technologies for rapid screening and analyses of biosystems are reviewed. Strategies to improve productivity *via *metabolic and integrated engineering are also highlighted.

## INTRODUCTION

Findings in the 1950s that DNA is the molecule that encodes proteins, which in turn control all the cellular processes inside the organism, have provided the impetus for the biotechnology era [1]. This has led to the advent of recombinant DNA technology and hybridoma technology in the 1970s, which marks the birth of modern biopharmaceutical development. As far as drug discovery and development is concerned, this is a significant milestone as some molecules are too complex and far too difficult to be extracted and purified from living materials, or synthesized chemically [2]. Genetic engineering provides an alternative means for the production of therapeutic proteins through the use of bacteria, yeasts, insect, animal and plant cells. The compounds produced provide alternative therapies for serious life threatening diseases such as cancer, viral infection or hereditary deficiencies, and other untreatable conditions [1].

Various technologies have since emerged ranging from the innovations in broad-based, rapid screening and macroscale analyses, to the sophistication in the imaging, control and automation technologies. Also contributing to the rapid progress are the innovations in gene therapies, antisense, cell surface engineering and molecular diagnostics. The production of biopharmaceuticals *via *recombinant technologies has led to new, innovative products, as well as significant improvements in quality and yield of existing products. They are better defined scientifically, with consistent quality and are free from infectious agents due to stringent cGMP guidelines [2]. The industrial scale manufacturing of penicillin G by the fermentation of mould *Penicillium* *notatum* in the early 1940s is the early success story of the use of living cells for drug production to combat infection by *Staphylococcus *and other bacteria [3]. Since mid-1970s, large scale production of hundreds of therapeutic proteins such as insulin, monoclonal antibodies, interferons or interleukins, have been developed [1,2,4].

The world wide pharmaceutical market is estimated to grow to $1.3 trillion by the year 2020 [5]. While chemical-based drug continues to be the major source of drugs, the world-wide biopharmaceutical market in 2003 is estimated in the region of $30-35 billion, accounting for 15% of the overall world pharmaceutical market [6]. Of these, plant-derived drugs and intermediates account for approximately $9-11 billion annually in the USA [2,5]. It was the scientific and technological innovation in drug discovery and development that had led to the creation of hundreds of start-up biopharmaceutical companies in the 1970s and 80s. With the basic research done in the universities and research institutions, the synergies between industrial players and academia over the years have resulted in the new technologies and tools to find new molecules to combat diseases; development of methods and biomarkers for clinical phenotyping; and validation of biochemical hypothesis of a drug candidate [1,2,4]. The growing confidence and interest in biopharmaceuticals has pushed big pharma companies to acquire technologies or invest in manufacturing facilities. Merck has bought RNAi developer, Sirna Therapeutics for $1.1 billion (RNAi being short interfering molecules to inhibit any gene of interest in any cells) [7]. Genentech has invested $140 million to set-up microbial-based manufacturing operations for biotherapeutics in Asia [8]. Despite high expenses in R&D, Merck and Genentech earn $32.8 and $1.4 billion in revenue, respectively, in the year 2000 [4]. This review article examines the practices, developments, and future trends in the production of biopharmaceuticals.

## HOST SYSTEMS FOR MOLECULAR PHARMING

The triggering factor behind the revolution in biopharmaceutical industries can largely be attributed to the development of advanced methods in the field of recombinant DNA technology. Cell engineering and transgenic technology border on several enabling techniques in such diverse fields as cell biology, embryology, molecular genetics, bioprocesses and metabolic engineering. A more directed approach to improve the cells or a given pathway of interest have become possible with specific genetic perturbations through modification of the promoter strength of a given gene, or by gene deletions, or by introducing whole new genes or pathways into the cells [9-12]. This means that the alteration effects can be determined and the amount directly-probed and produced at a specified quantity.

Scientific advances gained by transgenic capabilities also enable further understanding of basic biological pathways and yield insights into how changes in fundamental processes can perturb programmed development or culminate in disease pathogenesis [13]. Primary step behind recombinant DNA technology is the introduction of heterologous gene(s) in a non-native genetic background, and the sufficient expression of a cloned gene in the new host system. Two types of gene library are known, namely the genomic library which includes all the total chromosomal DNA of an organism; and cDNA library which corresponds to the mRNA fraction from a cell or tissue at specific point and time [14].

### Recombinant Microorganisms

Biopharmaceuticals produced from microorganisms that have gained marketing approval are invariably produced in the recombinant *E. coli* cell systems such as *E. coli* K12. The species are well-studied, documented and optimized as hosts for gene cloning [15-17]. These biopharmaceutical products include tissue plasminogen activator, insulin, α,γ-interferons, interleukin-2, granulocyte colony stimulating factor and human growth hormone [1,2,18]. The advantages that are normally associated with *E. coli* as the source of biopharmaceuticals include the well-characterized molecular biology and the ease of genetic manipulation; high levels of heterologous protein expression (as high as 25% of total cellular protein in the case of γ-interferon); relatively simple and inexpensive media, supported by well-established fermentation technology [2].

However, vast bulk of heterologous proteins from *E. coli* are intracellular which could complicate the downstream processing. There is also the possibility of the formation of a highly-densed inclusion body or insoluble aggregates of proteins, which could overload the normal protein-folding mechanism. Being a gram negative bacteria, the presence of endotoxin molecule lipopolysaccharides (LPS) in *E. coli* is a concern [2,19]. LPS, which make up 75% of *E. coli*’s outer membrane surface, influence the hypothalamic regulation of body temperature when they enter the blood-stream, and cause fever, which in some instances fatal. *E. coli* also often does not recognize the upstream elements of genes derived from different bacterial genera or families, or in some cases the over expressed protein may be toxic. Yeast protein kinase over-expressed in *E. coli* for example has been found inactive, but the same protein is active when expressed in yeast [20]. It is therefore common to transfer a gene which is originally cloned in* E. coli*, back into its native genetic background such as the α-amylase gene cloned in *E. coli* but transferred and expressed in its native *Bacillus amyloliquefaciens*, or antibiotic biosynthetic genes in *Streptomycetes* species [2].

Many therapeutically useful proteins, when naturally produced in the body are glycosylated. The limitation in biological activities of some expressed proteins from prokaryotes has been attributed to their limited capability to carry out post-translational modifications (PTMs) [2,21]. Vast majority of therapeutic proteins undergo several PTMs, which are the final steps in which genetic information from a gene directs the formation of a functional gene product. PTMs include covalent modifications of individual amino acids residues such as glycosylation, phosphorylation, methylation, ADP-ribosylation, oxidation and glycation; proteolytic processing and non-enzymatic modifications such as deamidation and racemisation. Most therapeutic proteins require at least proteolytic cleavages, oligomerization and glycosylation for their bioactivity, pharmacokinetics, stability and solubility [22,23]. Polyglutamylation is an example of PTM that generates lateral acidic side chains on proteins by sequential addition of glutamate amino acids. This modification is first discovered on tubulins, and is important for several microtubule functions [24]. As complex therapeutic proteins produced in prokaryotes are not always properly folded to confer the desired biological activity, microbial expression system is suitable mainly for the expression of relatively simple proteins which do not require folding or PTMs to be biologically active. Table **1** shows the comparison between different transgenic systems for the production of recombinant proteins.

### Eukaryotic Cell Systems

Major advantage of eukaryotic expression systems such as yeast, Chinese hamster ovary strain K1 (CHO-K1) or Baby hamster kidney cells (BHK), is their ability to carry out PTMs of protein product. While bacteria and yeasts may only be suitable for the synthesis of antibody fragments, insect cells infected by baculovirus and CHO cells can be the source of intact antibodies. Animal cell cultures are being used for the production of monoclonal antibodies *via *hybridoma cell technology; and vaccines production such as yellow fever viral particles *via *chick embryos culture, hepatitis A viral vaccines *via *human diploid fibroblast or gp120 [2]. Fungi such as *Aspergillus niger* and yeast such as *Pichia pastoris* have received considerable attention due to their potential for high level of protein expression such as factor VIII, α-interferon or Human Serum Albumin; and protein is excreted out into the extracellular media [25-27]. The gp160 HIV vaccines for example have been produced not only in insect or CHO cell-lines, but also in *Saccharomyces cerevisiae* [2]. Large scale production of polypeptides normally present on the surface of pathogen can now be produced by producer organisms such as hepatitis B surface antigen (HBsAg) genes expressed in yeast for the production of clinically-safe subunit vaccines [28].

Yeast has received considerable attention due to its more detailed genetics and molecular biology that can facilitate genetic manipulation, and a long history of application in brewing and baking. It is thus considered as GRAS organism (Generally regarded as safe) [2,29,30]. Important advantage for the use of yeast as host system is its ability to assemble DNA fragment in genomes by homologus recombination which allows the insertion of DNA sequences at specific locations in the yeast genome [31]. In the application of “biodrug” into the gastrointestinal tract, yeasts can be advantageous over bacteria, for the functional expression of heterologous genes, and they are not sensitive to antibiotics with high level of resistance to digestive secretions [30,32]. A “biodrug” concept involves the use of recombinant microorganisms as new delivery vehicles which may have potential medical applications in the correction of enzyme deficiencies, the control of the activation of pro-drug to drug or the production of therapeutic proteins, such as vaccines, directly in the digestive tract. The recombinant cells may produce active compounds such as hormones, enzymes, and vaccines; or perform bioconversions or “biodetoxication” [32,33].

The eukaryotic cell-based system however may need a more complex nutritional requirement as compared to *E. coli*. It may also require a carefully controlled-fementation condition due to the shear sensitivity of the cells, and also to control protein glycosylation which may depend on cell metabolism such as that in CHO [34]. Cell growth are much slower, the post-translational glycosylation pattern especially in yeast and fungal cells may be inappropriate or different from the pattern observed in native glycoprotein isolated from natural sources, and the cells are often proned to contamination by virus or prions [2]. Scaling-up of cultured mammalian cells to large volumes is more difficult. It may take 4 years to build a 100000L fermenter, costing $400 million for CHO cells [35]. Because of huge capital costs, industry has been unable to keep up with the growing demand [36]. Another method that can be explored is to extract biopharmaceuticals from animal and human tissues such as insulin from pig and cow pancreas, or blood proteins from human blood [37]. However, these also incur high-cost and carry the risk of transmitting infectious diseases to humans [38].

### Transgenic Animal

The biochemical, technical and economic limitations of prokaryotic and eukaryotic expression systems have spurred interest in transgenic animal and plant as new expression systems. Transgenic animal as a bioreactor system for pharmaceutical production, or for modification of tissues and organs for transplantations, or as a model system from DNA microinjection to gene targeting and cloning, has had a significant impact on human health, pharmaceutical discovery and the drug pipeline [13]. Transgenic modifications, particularly in mice, are commonly used to model human conditions. The use of transgenic animal have been beneficial in the studies for drug discovery in human developmental and pathological conditions, including gene therapy, genetic basis of human and animal disease, the assessment of the validity of therapeutic strategies before clinical trials, disease resistance in humans and animals, drug and product testing or toxicological screening, and novel product development through molecular pharming [13,39].

The production of therapeutic proteins from transgenic animals involves the expression from mammary-gland specific promoters to drive secretion of the transgene into milk, or the use of kidney- or bladder specific promoters that direct transgene expression to the urine [40-42]. Mammary specific expression is achieved by fusing the gene of interest with promoter containing regulatory sequences of a gene coding for a milk-specific protein, such as whey acid protein promoter in the β-casein and α- β-lactoglobulin genes [2]. The early success story of transgenic expression of a Human Tissue-Type Plasminogen Activator (tPA) has been reported in mouse milk [43] and Goat Milk [44]. The European Medicines Agency (EMEA) has approved the use of ATryn, a drug extracted from the milk of goats engineered to carry a human gene involved in inhibiting blood clots [45]. For monoclonal antibodies, the vast majority of production source are of murine origin [2]. Major problems associated with murine antibodies include the reduced stimulation of cytotoxicity and the formation of complexes after repeated administration, resulting in mild allergic reactions and anaphylactic shock [46]. Rabbit milk is seen as an attractive alternative source for antibodies, as rabbit is not susceptible to prion diseases and is known to transmit only rare and minor diseases to human [47]. Other various end-organs and system being investigated for antibodies production include blood, urine and other tissues, and egg white from transgenic chicken. In 2002, a mature and functional human immunoglobulins has been reported in the blood of transchromosomic calf. This is achieved by introducing a human artificial chromosome (HAC) vector containing the entire unrearranged sequences of the human immunoglobulin (h*Ig*) heavy-chain (*H*) and lambda (λ) light-chain loci, into bovine primary fetal fibroblasts using a microcell-mediated chromosome transfer (MMCT) approach [48]. The use of the transchromosomic calves is an important step in the development of a system for producing therapeutic Human polyclonal antibodies (hPABs).

In the area of human xenotransplantation, the transgenic models remain a viable option in dealing with severe donor organ shortages. Research continues to address the biological barriers with regards to hyperacute rejection mediated by preformed natural antibodies and complement [13,49]. An important development in the area of xenotransplantation is the stem cell and nuclear transfer cloning procedures such as that being developed in the production of α-1,3-galactosyltransferase knockout pigs [50-52]. The embryonic stem-cell technology has moved from the well studied transgenic mouse to now include the transgenic fish, chicken, rabbits, sheep and cattle [53,54]. The main advantages of transgenic animal as a source of biopharmaceutical production are as shown in Table **1**. High protein expression level is achievable (in many cases exceeding 1 g protein/litre milk, which may be similar to 50-100 litre bioreactor in a day) [2]. There is less environmental concern as transgenic farm animals are kept in enclosed areas. The only drawback is the variability in the expression levels ranging from 1 mg/L to 1 g/L. This can be improved through vector optimization and the use of gene insulators for increased and more predictable production such as for antibodies in milk [47,53].

### Transgenic Plant

Both transgenic animal and plants may be the only tools capable of producing high level of protein or antibodies [47]. Different types of therapeutic proteins such as blood and plasma proteins, vaccines, hormones, cytokins and growth factors, enzymes and others such as hirudin, endostatin and human lactoferrin, have been produced in transgenic plant systems mainly in tobacco and potato [22]. Many antibodies or antibody fragments have been produced for therapeutic or diagnostic purposes in various plant expression systems [55]. These plant-based antibodies are correctly assembled, proteolytically matured and glycosylated, with high mannose and biantennary complex type *N-*glycans [56,57]. While animal and plant cells may have similar capacity to assemble antibody subunits, plants may differ from animals in carrying out PTMs, as far as the capacity to glycosylate antibodies is concerned. This is pertinent as glycosylation is required for stable antibodies *in vivo* and in inducing complement and antibody-dependent cellular cyotoxicity (ADCC) [47]. Despite differences in *N-*glycan structures, antibodies produced in plants have similar antigen-binding capacity as their homologs produced in mammalian cells. But unlike mammalian cell cultures, plants are devoid of human infective viruses and prions [2,22]. Furthermore, an antibody half-life in the bloodstream as well as its ability to be recognized by Fc receptors, which are both determined by heavy chains N-glycosylation, are not strongly affected when a plant-N-glycan is present instead of a mammalian N-glycan [58-60]. Glycosylation of antibodies can be improved in plants and animals by transferring the genes encoding enzymes capable of adding *N*-acetylglucosamine (GlcNac), sialic acid, fucose and galactose to the *N-*glycans [47].

Current limitations of plant expression systems are low yields of some therapeutic proteins and the impact of non-mammalian glycosylation on the activity, immunogenicity and allergenicity of glycosylated plant-made pharmaceuticals. The *N-*glycan of antibodies extracted from plants (plantibodies) have been reported to not only unable to confer some biological properties and to induce immune response when tested in mice, but also may have undesirable side-effects in patients [47]. Different strategies and new plant expression systems are currently being developed to improve the yields and quality of plant-made pharmaceuticals. The challenges include in choosing the transformation systems, adaptation of codon usage, gene silencing, design of recombinant transgenes with appropriate expression, tissue specificity and proper developmental regulation, and subcellular localization of products [22]. The location of protein accumulation within the cell is important to ensure correct folding and stability of the protein [61]. Different plant organs (leaves, seeds, root) and plant cell compartments (endoplasmic reticulum, chloroplast, vacuole and oil body) have been used to express many therapeutic proteins [62,63]. In plants with large foliage volume such as tobacco, alfalfa and legume plants, expression is performed in leaves. In potato, corn, rapeseed, safflower, soybean, wheat or rice, protein accumulation is achieved in tubers or in seeds [64,65]. In plants, genetic material is distributed between the nucleus, plastids, and mitochondria. Traditionally, the production of transgenic plants, for basic and applied purposes, has been mainly through transgenes expression in the nucleus. Nonetheless, there is a concern that transgenes may escape and contaminate the environment *via *pollen such as in corn [66]. There is now an increasing interest in expressing the transgenes in chloroplasts rather than the nucleus as the genes expressed in the plastome will not be transferred through pollination [66,67].

Most biopharming applications target production and storage in seeds, which naturally accumulate high concentrations of proteins and oils, and the easiest part of the plant to store and transport to processing facilities. Two seed-specific “promoters” have been used experimentally - the beta-phaseolin promoter of common bean and the oleosin promoter of *Brassica* species [61]. Recent advances in the control of post-translational maturations in transgenic plants will allow them to perform human-like maturations on recombinant proteins and make plant expression systems suitable alternatives to animal cell factories [22]. Antibodies that currently cost thousands of dollars per gram might be produced in plants for $200 per gram [68]. Transgenic plants are expected to produce up to 10 kg antibodies per acre [69] or 1 kg of plantibodies after 36 months. This may be achievable with rabbit milk, but not goat milk [58]. A plant “bioreactor” will allow the production of recombinant proteins up to 20 kg/ha, regardless of the plant material considered – tobacco, corn, soybean or alfalfa [58]. With the whole gamut of issues pertaining to potential host systems for recombinant protein production elaborated and recognized, in the next part, new development and future direction of research in biopharmaceuticals production are discussed.

## SCIENTIFIC AND TECHNOLOGICAL INNOVATIONS IN BIOPHARMACEUTICALS

### Human Stem Cells

The discovery that cells are capable of self-renewal has led to the functional definition of stem cells [70,71]. This has great impact in the area of targeted therapies and drug delivery as human stem cells may not only find application in the repair, regeneration and cellular replacement of damaged or defective tissues, but also in the toxicological screening and discovery of new therapeutic drug molecules, and as a tool for *in vitro* investigation of cellular and developmental processes [72-74]. Human stem cells can be isolated, purified, expanded in number and differentiated into the cell type of choice in a controlled manner. The cells may be sourced or derived from blood and tissues postnatally (‘adult’ stem cells), and from the fetus (fetal stem cells) or from the blastocyst in the developing embryo prior to implantation (embryonic stem cells) [72,75]. Adult stem cells and progenitor cells found in adult tissues, act as a repair system for the body, replenishing specialized cells, and also maintain the normal turnover of regenerative organs, such as blood, skin or intestinal tissues. They have been used to treat successfully leukemia and related bone/blood cancers through bone marrow transplants [76].

Of great interest today is in the derivation and culture of Human embryonic stem cells (hESC). These cells are pluripotent and undifferentiated, and can grow *in vitro* indefinitely. They can potentially provide a supply of readily available differentiated cells and tissues of many types to be used for therapeutic purposes, as well as for drug screening and discovery [77]. The actual methods of hESC derivation have not changed greatly since the method first porposed [72,77]. It requires establishment of embryonic stem cell lines using the inner cell mass of an early pre-implantation embryo or excess human embryos from *in vitro* fertilization treatments. To enable the clinical use of hESC for cell transplantation, the use of animal derived biological components is no longer acceptable. The main emphasis over the last several years has been in finding defined conditions for derivation and culture of hESCs. The aim is to replace even human derived materials with completely defined systems. The use of embryonic stem cells is embroiled with ethical issues as the blastocyst may be destroyed during the process [72,77,78]. There are also challenges to generate cells of sufficient quality and quantity and to expand cell numbers while maintaining the fidelity of phenotype. Strategies need to be developed to control and direct differentiation to produce the cell type of interest in a format that is suitable for intended purposes [79].

### Gene and Targeted Therapies

Gene therapy is a novel technique, emerge as a direct result of recombinant DNA revolution. Though still highly experimental, it has the potential to become an important treatment regimen as it allows the transfer of genetic information into patient tissues and organs for the diseased genes to be eliminated or their normal functions rescued. The procedure allows the addition of new functions to cells, such as the production of immune system mediator proteins that help to combat cancer and other diseases [80]. The technique entails stable introduction of a gene into the genetic complement of a cell, such that subsequent expression of the gene achieves a therapeutic goal. The desired gene can be naked DNA as in the case of DNA-based vaccine; or packaged into a vector system such as retroviruses or plasmid-containing liposomes, or microencapsulated, to affect gene transfer [2,81-83]. Once assimilated by the cells, the exogeneous nucleic acids must be delivered to the nucleus. The *in vitro* approach necessitates removal of the target cells such as blood cells, stem cells, epithelial cells, muscle cells or hepatocytes from the body, cultured *in vitro* together with vector containing nucleic acid to be delivered, and the genetically altered cells reintroduced into the patient’s body. Another approach is direct administration of the nucleic-acid containing vector to the target cell, *in situ* in the body, such as direct injection of vectors into a tumour mass, or aerosol administration of vectors containing cystic fibrosis gene to respiratory tract epithelial cells. For intravenous injection, vector can be designed to be bio-specific such that it will recognize and bind only to the specified target cells. Selective delivery can be made possible by the inclusion of antibody on the vector surface, which specifically binds a surface antigen uniquely associated with the target cell; or vector engineered with a specific hormone that can only bind to cells displaying the hormone receptor [2]. The therapeutic potential of gene therapy includes curing in-born errors of metabolism, or conditions induced by the presence of a defective copy of a specific gene (s), including cancer, AIDS, cystic fibrosis, haemophilia, neurological disorder, or retinal degeneration [2,84-86].

The antisense approach is a type of gene therapy based upon the generation of short, single-stranded stretches of DNA or RNA, termed antisense oligonucleotides, displaying specific nucleotide sequences. These oligonucleotides can be synthesized and are capable of binding to DNA at specific gene sites or mRNA derived from specific genes. The translation of mRNA can be blocked, preventing the synthesis of mature gene protein product. This may have potential application in treating disease states which require blocking of gene expression for curing effect [2]. However, antisense DNA technology has failed to live up to its early hype, as most standalone antisense companies folded [7]. The downfall of antisense has been attributed largely to its off-target effects, especially the tendency of nucleic acid sequences to induce generalized immune responses, as well as the difficulty of delivering the therapies to the right cell types.

Another nucleic acid-based therapy, short interfering RNA (siRNAs) or microRNAs (miRNAs) or RNAi therapies, have been hailed as the hallmark of new frontiers in biotechnology [7]. These are small noncoding RNAs that regulate gene expression by repressing translation of target cellular transcripts. Though the extent of miRNA regulation is not well known, increasing evidence indicates that this is a naturally occurring mechanism in eukaryotes, and miRNAs have distinct expression profiles and play crucial roles in numerous cellular processes. miRNAs can induce a cell to destroy complementary pieces of mRNA, preventing the target message from being transcribed. This RNA inhibition can be exploited to inhibit any gene of interest and may be particularly useful in gene therapies [2,7,87]. The major challenges with developing RNAi therapies also involve delivery to the target site, and cross-talk in signaling pathways. Transgene expression can be suppressed in hematopoietic cells using vectors that are responsive to miRNA regulation. A study has shown that by challenging mice with lentiviral vectors encoding target sequences of endogenous miRNAs, the efficiency of miRNAs is achieved in sharply segregating the gene expression among different tissues [87]. In another study, analysis of the relationship between miRNA expression levels and target mRNA suppression suggests that the suppression depends on a threshold miRNA concentration, which makes it pertinent to exploit the miRNA regulatory pathway and to generate vectors that could rapidly adjust transgene expression in response to changes in miRNA expression [84]. The properties of miRNA-regulated vectors should allow for safer and more effective therapeutic applications [88]. This fast target validation and easy synthesis of miRNAs are attracting attention from drug developers, as a potential multi-billion dollar therapeutic platform in the next few years [7].

### Metabolic Engineering

The approaches used to improve foreign-protein production in various expression systems include strain improvement by mutagenesis and screening and genetic modifications such as the deletion of proteases from the production strain, the introduction of multiple copies of expressed genes, the use of strong promoters, gene fusions to well-secreted proteins, the use of native signal sequences, and overexpression of individual endoplasmic reticulum-resident genes [89-92]. The basic idea is for increased productivity, cost reduction, and for developing new strains with more specific desirable characteristics such as achieving a more complete PTMs that could accentuate, diminish or eliminate the activity of the desired enzyme. This may require a more comprehensive analysis of the recombinant organism in terms of its biology, kinetics, physiology and performance.

Metabolic engineering takes strain improvement, from empirical approach through mutagenesis and selection, to a more directed improved productivity through the modification of specific biochemical(s) or the introduction of new one(s), *via *molecular biology, physiology, bioinformatics, computer modeling and control engineering [9-12]. Metabolic engineering comprises a synthesis step that introduces new pathways and genetic controls; an analysis step to elucidate the properties of metabolic reaction networks in their entirety; and the evaluation of the recombinant physiological state *via *metabolic flux determination [11]. The way and the speed of improving biosystems is greatly changed through this systems-based analysis as it involves identifying the reaction and/or transport bottlenecks, thermodynamic feasibility, pathway flux distribution and flux control.

Metabolic engineering has already made impact in drug discovery through the synthesis of enhanced or novel natural products and proteins such as carotenoids, ascorbic and lactic acids, xylanases, progestrones, amino acids and novel precursors to amino acids, biopolymers and chiral chemicals, extension of substrate range for growth and product formation, and the detoxification, biodegradation or mineralization of toxic pollutants [93]. The metabolic engineering approach in the precursor formation of useful stereochemistry has been demonstrated in the bioconversion of indene to 1-indenol and cis-(1S,2R)-indandiol, from which Cis-aminoindanol, a key chiral precursor to the HIV protease inhibitor CRIXIVAN, can be derived. A new operon encoding a toluene-inducible dioxygenase (TID) discovered from *Rhodococcus sp.* I24 strain, has shown the capability of converting indene to 1-indenol and cis-(1S,2R)-indandiol, when the operon is heterologously expressed in *E. coli* [94].

### Genomics, Transcriptomics, Proteomics, Metabolomics and Fluxomics

The synthesis aspect in metabolic engineering can be achieved if the genes to be expressed are available, but analysis is more of a problem, due to the the complexity of the cellular metabolism and the lack of a breakthrough technology to deal with it [11,95]. A number of powerful techniques have been developed that enable a far more in-depth analysis of the cellular physiology. These include DNA sequencer for genomic analysis, DNA microarray for transcriptomic analysis (simultaneous quantification of all gene transcripts in a cell), two-dimensional gel electrophoresis, protein microarray and protein function microarray for proteomic analysis (simultaneous quantification of a large number of proteins in a cell), gas chromatography (GC), high performance liquid chromatography (HPLC), nuclear magnetic resonance (NMR), direct injection mass spectrometry (MS), or Fourier transform infrared (FT-IR) and Raman spectroscopy for metabolomic analysis (analysis of the intracellular metabolite levels), advanced fermentation technology with on-line control and monitoring, and bioinformatics (including mathematical models for analysis of pathway structures and control of pathway fluxes) [95-97]. Advances in sequencing and DNA replication technologies have made it possible to sequence the entire genomes of many organisms. Genomes of more than 170 microorganisms have been completely sequenced and more than 190 sequencing projects were active in July 2004 [98]. The completion of these sequencing projects coupled with rapid development of genome-derived technologies will spur the effort towards linking the global gene expression analysis with cell physiology. Of greater impact in this effort is the development of protein microarrays and protein function arrays which have been suggested to have greater diversity of assays than DNA microarrays. A novel type of protein array has been developed where the recombinant proteins are bound to the surface without possible losses of functions. The first yeast proteome chip investigating protein-protein interactions and lipid-binding; and a human proteome chip composed of 5000 proteins have been reported [99].

Another indispensable area in metabolic engineering for direct pathway modification and strain analysis is metabolomics analysis. Metabolomics offers the unbiased ability to differentiate organisms or cell states based on metabolite levels that may or may not produce visible phenotypes/genotypes. To understand the global cellular functions at multi-controlling steps, it is imperative to carry out combined analysis of transcriptome, proteome and/or metabolome simultaneously [100]. In comparing two different strains of *E. coli*, it has been established through metabolic flux, NMR/MS and Northern blot analysis, that the glyoxylate shunt, the TCA cycle, and acetate uptake by acetyl-CoA synthetase are more active in *E. coli* strain BL21 than in JM109. This has resulted in the differences in the glucose metabolism and acetate excretion. Upon closer examination with microarrays and time course Northern blot, it is found that not only the glyoxylate shunt, the TCA cycle and the acetate uptake are different, the other metabolic pathways such as gluconeogenesis, anaplerotic *sfcA* shunt, *ppc* shunt, glycogen biosynthesis, and fatty acid degradation, are also active differently in the two strains [101].

### Bioinformatics

The advent of bioinformatics through genome databases, protein databanks and other databases containing detailed information about biological systems has changed the landscape of biological and bioengineering research. This rapid growth of bioinformatics databases and pattern discovery methods provides a powerful means of achieving the goals of metabolic engineering. The combination of computational biology and expression-based analysis of large amounts of sequence information emerge as indispensable tools for gene discovery and characterization. The paradigm has changed from ‘vertical’ analysis of the role(s) of one or a few genes to ‘horizontal’ holistic approaches, studying the function of many or even all of the genes of an organism simultaneously [96]. This requirement, coupled with the rapidly increasing database size necessitates pattern recognition algorithm for association formation, feature extraction, and identifying homogeneous subsets of data with similar characteristics or dominant discriminating characteristic that can be used for sequence identification, function assignment or process diagnosis [102]. Several pattern recognition algorithms have emerged which has proven effective in identifying patterns across all data sets. These include Principal Component Analysis, Cluster Analysis, Mean Hypothesis Testing, multi-resolutional scale analysis by Wavelet Transforms, Decision Trees, and Artificial Neural Networks.

Gene prediction or gene finding is the most important step to understand the genomic species once it has been sequenced [103]. The individual genes can be algorithmically identified from the stretches of sequence, usually genomic DNA; while the biochemical function of a gene can be deduced by comparing the DNA sequence with the sequences of genes of known function in the databases. A facility where researchers can interactively scan for recurring patterns in the sequence, or investigate reading frames, variable regions, positions of particular codons could facilitate quick overview of the sequence features [104]. New computer tools may prove indispensable to compose genetic data at all levels of biological organization - from gene to population, species and ecosystems - for multiple purposes, including gene conservation. Similarly, structural genomics projects have begun to produce protein structures with unknown function. Such progress requires accurate, automated predictors of protein function to be developed if all these structures are to be properly annotated in reasonable time. Algorithms that can align more than two sequences (i.e., multiple alignments) can help elucidate phylogenetic relationships within protein families, thus providing new insight into the evolution of a protein and its potential utility [105,106]. Identifying the interface between two interacting proteins provides important clues to the function of a protein and can reduce the search space required by docking algorithms to predict the structures of complexes. An increasingly popular machine-learning approach, the support vector machine (SVM), has been applied for protein–protein binding sites prediction with high accuracy whilst avoiding over-fitting, using the profiles of spatially and sequentially neighbouring sequences and also sequence neighbours of a target residue. SVM have also found applications in gene expression classification, protein classification, protein fold recognition and prediction of protein solvent accessibility, β-edge strands, single nucleotide polymorphisms, protein secondary structure, protein quaternary structure and T-cell epitopes [106].

Another important area is in protein–protein interfaces and development of methods for predicting protein interface residues. The side chains of the amino acids, owing to a large extent to their different physical properties, have characteristic distributions in interior/surface regions of individual proteins and in interface/non-interface portions of protein surfaces that bind proteins or nucleic acids. These distributions have important structural and functional implications [107,108]. Interface prediction methods rely on a wide range of sequence, structural and physical attributes that distinguish interface residues from non-interface surface residues. The input data are manipulated into either a numerical value or a probability representing the potential for a residue to beinside a protein interface. Accurate methods have been developed for predicting the solvent accessibility of amino acids from a protein sequence and for predicting interface residues from the structure of a protein-binding or DNA-binding protein [107]. Satisfactory predictions for complex-forming proteins that are well represented in the Protein Data Bank have been achieved, but less so for the under-represented ones. Efforts are reportedly being made in building structural models for multi-component structural complexes [108].

### High Throughput Bioprocess Development

**Fig. (1):**
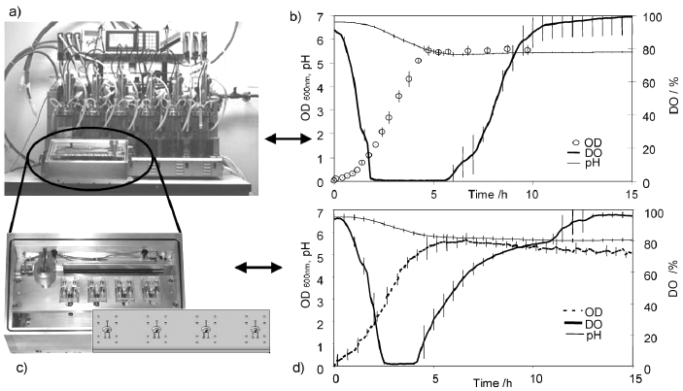
**(a)** Multiplexed microbioreactor system. The “Sixfors” bioreactor system containing six bench-scale reactors (Infors, Switzerland). **(b)** Fermentation data obtained with the Sixfors. **(c)** The multiplexed system with four stirred microbioreactors and an integrated microbioreactor cassette. **(d)** Fermentation data obtained with the multiplexed microbioreactor system [112] *(Reproduced by permission of The Royal Society of Chemistry).*

With greater need for rapid sampling and accurate information on the interactions between biosystems and the bioprocess operations, microfabrication and array-based testing could revolutionize the drug discovery process. Miniaturized analytical devices could reduce reagents and sample consumption, and improve the analytical speed by reducing the time required by running several analyses in parallel [109]. In combination with optical sensor technology, low-cost microbioreactor is relevant to investigate biological kinetics and for high-throughput evaluation of the operational or nutritional parameters on cell growth and product formation in a systematic and statistically significant manner [110,111]. A multiplexed microbioreactor system with a working volume of 150 μl for simultaneous operation of up to eight microbioreactors has been reported (Fig. **1**) [112]. The reactors, fabricated of poly(methyl methacrylate) (PMMA) and poly(dimethylsiloxane) (PDMS), include miniaturized motors for magnetic stirring of the reactors, and optic sensors for measuring the fermentation parameters. Optical density is determined with a transmittance measurement through the reactor chamber, and *in-situ* measurements of dissolved oxygen and pH are obtained with fluorescence lifetime sensors embedded in the bottom of the reactor chambers. The multiplexed microbioreactor system monitors and records the process parameters in real time for each microbioreactor. Parallel batch culture of *E. coli* fermentation data of cell growth, DO, and pH compare favorably with microbial fermentations undertaken with the same strain and under the same conditions in multiple bioreactor systems at the bench scale. In another system, a well-mixed, 150 μl, membrane-aerated microbioreactor run in chemostat mode reach a steady state condition at which *E. coli* cell biomass production, substrates and the product concentrations have been reported to remain constant. The reactor is fed by a pressure-driven flow of fresh medium through a microchannel. Chemotaxisial back growth of bacterial cells into the medium feed channel is prevented by local heating. Using poly(ethylene glycol) (PEG)-grafted poly(acrylic acid) (PAA) copolymer films, the inner surfaces of PMMA and PDMS of the reactor wall are modified to generate bio-inert surfaces resistant to non-specific protein adsorption and cell adhesion. These modified surfaces effectively reduce wall growth of *E. coli* for a prolonged period of cultivation [113].

**Fig.(2):**
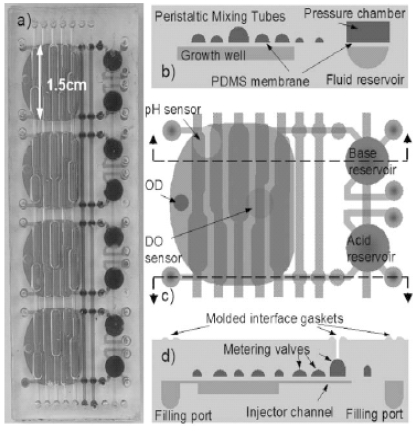
Microbioreactor array module. **(a)** Four reactors integrated into a single module. **(b)** Cross-section of a microbioreactor showing the peristaltic oxygenating mixer tubes and fluid reservoir with pressure chamber. **(c)** Top view of a microbioreactor showing optical sensors and layout of peristaltic oxygenating mixer and fluid injectors. Growth well is 500 mm deep, with a 100 μl working volume. **(d)** Cross-section showing the fluid injector metering valves [114] *(Reproduced by permission of The Royal Society of Chemistry).*

An integrated array of microbioreactors has also been developed, leveraging on the advantages of microfluidic integration to deliver a disposable, parallel bioreactor in a single chip, rather than on robotically multiplexing independent bioreactors. The system offers small scale bioreactor arrays with the capabilities of bench scale stirred tank reactors. The microbioreactor with 100 μl working volume, comprise a peristaltic oxygenating mixer and microfluidic injectors (Fig. **2**). These integrated devices are fabricated in a single chip and can provide a high oxygen transfer rate (*k_L_a* ≈ 0.1 s^-1^) without introducing bubbles, and closed loop control over dissolved oxygen and pH (± 0.1). The system reportedly could support eight simultaneous *E. coli* fermentations to cell densities greater than 13 gDW/L comparable to that achieved in a 4 L bench scale stirred tank bioreactor. This is more than four times higher than cell densities previously achieved in microbioreactors. Bubble free oxygenation permitted near real time optical density measurements could be used to observe subtle changes in the growth rate and infer changes in the state of microbial genetic networks [114].

### Rational Drug Design

High-throughput screening (HTS) involves screening of thousands to millions of compounds to identify target or lead compound with useful biological activities, and accessing the libraries of pharmaceutical and chemical companies. Such technique of blind-screening of millions of compounds in the lab and hoping for a hit or a lead has increasingly be seen instead as an irrational approach. Rational drug design which is synonymous with structure-based design, draws the emphasis away from traditional random screening. It involves a logical, calculated approach, which may include ligand-based approach to discovery [2,115]. It relies heavily upon computer modeling to modify an existing drug or design a new drug which will interact with selected molecular target important in disease progression. *In silico* methods are becoming more efficient as they allow scientists to hone in on and manipulate specific molecular structures of interest. A pre-requisite is the three dimensional structure of the drug’s target be known to ease the finding of the molecules that would interact more efficiently in an active protein site, and subsequently assist the chemists to design more efficient drugs [2,116]. Targets are normally proteins such as specific enzymes or receptors for hormones or ligand that would modify the target activity. An example being the activity of retroviral reverse transcriptase as an effective AIDS therapeutic agent. Predictive computer modeling software allows generation of a likely 3D structure from the amino acid primary sequence. This however must be complemented by X-ray crystallography to determine the exact 3D structure. Once the 3D structure of the target protein has been resolved, molecular modeling software facilitates rational design such as a small ligand capable of fitting into a region of an enzyme’s active site [2].

The development of combinatorial libraries, through techniques capable of generating large numbers of novel synthetic chemicals, coupled with high-throughput screening methods and the use of sophisticated knowledge-based approaches to drug discovery is becoming routine [2]. The discovery informatics software for virtual HTS (vHTS) will continue to play a vital role in rational drug design [115]. It has been suggested that without computer modeling, identification of a potent drug would require screening of hundreds of thousands of candidates, taking up to 10 years or so, costing hundreds of millions dollars. Computer modelling saves time and cost as the discovery can be made with a software, and fewer compounds to be prepared or modified to yield a highly effective drug; as compared to the cost in setting up an experimental HTS laboratory and developing assays to discover a compound [2,115]. Ironically, one of the major issues facing pharma and biotechs sector today is the lack of innovation. This downward spiral has been attributed among others to the heavy investment in computer-assisted drug design, in building chemical libraries and in high-throughput screening at the expense of hiring innovative chemists and biologists [117]. There are challenges in generating high quality protein crystals to facilitate X-ray analysis, as NMR can only determine 3D structure of small proteins [2]. The crystal structure does not always accurately depict how a molecule will behave *in vivo*; and the medicinal chemists also often find it difficult to develop new structures for the “rational” approach [116]. The “omics” technology coupled with efficient and effective “knowledge and disease management” strategies should offer new opportunities for achieving rationality in drug design. In addition, much drug discovery and development data requires the time dependence of biological responses, which means collecting the data at an infinite number of points, and employing time-series methods to give a clearer understanding of biological processes. With this new network biology era, it becomes pertinent for quantitative description of all the cellular communication networks and how they integrate. For these, validation of the networks through statistics to provide estimates of the robustness of the parameters and network structures; and identification and confirmation of the genetic regulation mechanism through fundamental genetics and biochemistry, are vital [116].

### Integrated Platform

Current research on chemical and pharmaceutical development and manufacturing for integrated systems focuses on advanced analytical and control techniques, computational methods for process invention and optimization and knowledge management. High-throughput microscopy and imaging analysis are becoming increasingly important with the development of fluorescence tagging, live cell experimentation, image acquisition and processing and computer software that brings all together. In pharmacotherapy, where there is a greater need to observe the changes in real-time, a microfluidic technology has been developed for highthroughput live-cell screening, with fluidic control techniques for kinetic studies, changes of media, changes of drugs or flow mixing, under microscopic scrutiny [118]. There is an increasing trend towards integration of *in situ* (on-line) spectroscopic measurements (particularly of reactions), real-time analysis of the spectroscopic signals, and feedback control to feeds, and dosing units in order to achieve desired reaction rates or selectivity. This is done with the implementation of chemometrics or multivariate statistical analysis for elucidating pertinent chemical information from various process analytical measurements [118]. Molecular imaging has become useful for drug discovery as there is a greater interest in understanding the mechanisms at the gene and molecular level. A new technology STORM (sub-diffraction limit imaging by Stochastic Optical Reconstruction Microscopy) has been developed where optical image is built through the orchestration of photon emissions of individual, switchable fluorescent molecules with molecular specificity for intracellular details. Another technique, MIMS (Multi-isotope Imaging Mass Spectrometry) takes advantage of the existence of stable non-toxic isotopes such as _15_N. Applications include in the pulse chase, small molecule drug target interaction and tracking the lineage of transplanted stem cells [119].

## CONCLUSIONS

The ultimate aim of biopharma development is to improve the quality of life and to extend longevity. The quest for new drugs is never ending, as is the need to understand disease causes beyond the symptoms. The rapid emergence of new technologies is revolutionizing the biopharma industry. As shown in Fig. (**3**), the approach in the development of biopharmaceuticals require multi-pronged strategies. Promising among these are the development of molecular diagnostic technologies to elucidate, evaluate and monitor diseases, vaccine technology principally the DNA-based viral vaccine, and the high-throughput screening platform with real-time monitoring and analysis. The future for biopharmaceuticals production is indeed extremely bright and offers an unprecedented opportunity.

## Figures and Tables

**Fig.(3):**
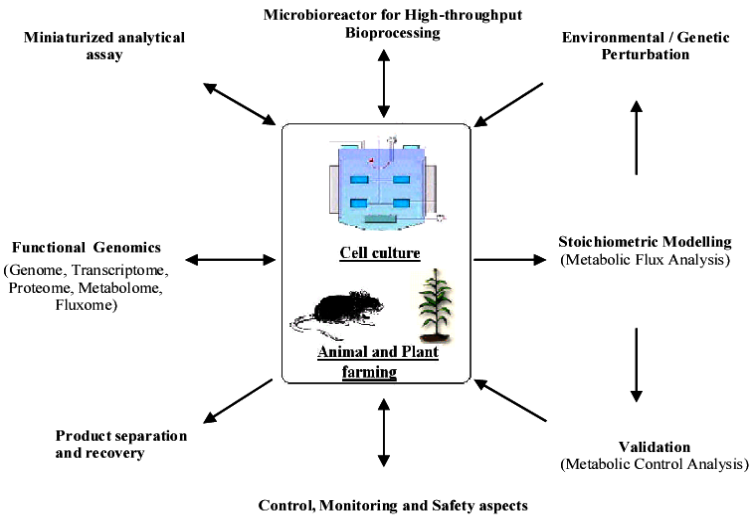
Biopharmaceuticals production considerations in a nut-shell (Modified from [120]).

**Table 1.** Transgenic Systems for the Production of Biopharmaceuticals

*Bacteria*	*Mammalian*	*Transgenic Animal*	*Transgenic Plant*
**1. Productivity** g/L culture **2. Advantages** Easy to culture Well-characterised **3. Limitations** Lack of PTMs Limited capacity **4. Issues** Presence of inclusion bodies, endotoxin molecules	g/L culture PTMs mechanism Inappropriate PTMs More complex media Slow cell growth Limited capacity Viruses and prions contamination	g/L milk or urine Low capital cost Easy to harvest Easy to scale-up PTMs mechanism Inconsistent product yield Methods for genetic modification	kg/ha or g/L (for latex) Low cost Easy to harvest Easy to scale-up PTMs mechanism Low product yield Environmental concern on GMOs Need stronger promoters Targeted protein expression in specific organs, cell compartment
